# EUS-Guided Vascular Interventions: Recent Advances

**DOI:** 10.3390/jcm13164835

**Published:** 2024-08-16

**Authors:** Sahib Singh, Saurabh Chandan, Sumant Inamdar, Kambiz S. Kadkhodayan, Jahnvi Dhar, Jayanta Samanta, Antonio Facciorusso

**Affiliations:** 1 Department of Internal Medicine, Sinai Hospital of Baltimore, Baltimore, MD 21215, USA; sahibs559@gmail.com; 2 Center for Interventional Endoscopy (CIE), Advent Health, Orlando, FL 32803, USA; saurabhchandan@gmail.com (S.C.); kambiz.kadkhodayan.md@adventhealth.com (K.S.K.); 3 Gastroenterology & Hepatology, University of Arkansas for Medical Sciences, Little Rock, AR 72205, USA; sumant.c.inamdar@gmail.com; 4 Department of Gastroenterology, Post Graduate Institute of Medical Education and Research, Chandigarh 160012, India; jahnvi3012@gmail.com (J.D.); dj_samanta@yahoo.co.in (J.S.); 5 Gastroenterology Unit, Department of Medical and Surgical Sciences, University of Foggia, 71122 Foggia, Italy; 6 Clinical Effectiveness Research Group, Faculty of Medicine, Institute of Health and Society, University of Oslo, 0372 Oslo, Norway

**Keywords:** EUS, vascular intervention, gastric varices, portosystemic pressure gradient, splenic artery embolization

## Abstract

Endoscopic ultrasound (EUS)-guided vascular interventions were first reported in 2000 in a study that evaluated the utility of EUS in sclerotherapy of esophageal varices. Currently, gastric variceal therapy and portosystemic pressure gradient (PPG) measurements are the most widely utilized applications. Ectopic variceal obliteration, splenic artery embolization, aneurysm/pseudoaneurysm treatment, portal venous sampling, and portosystemic shunt creation using EUS are some of the other emerging interventions. Since the release of the American Gastroenterological Association (AGA)’s commentary in 2023, which primarily endorses EUS-guided gastric variceal therapy and EUS-PPG measurement, several new studies have been published supporting the use of EUS for various vascular conditions. In this review, we present the recent advances in this field, critically appraising new studies and trials.

## 1. Introduction

Endoscopic ultrasound (EUS) has been widely adopted as a diagnostic and therapeutic tool for various gastrointestinal (GI) pathologies, ranging from minimally invasive biopsies to drainage procedures [1]. Using high-frequency waves, the ultrasound component enables clear visualization of the surrounding organs and blood vessels [2]. Given the close proximity of the GI tract to vascular structures, along with similar (or in some select cases) better success rates compared to interventional radiology (IR) and/or surgery, the position of EUS in vascular interventions is becoming stronger [3]. Originally described in 2000, when EUS-guided sclerotherapy was performed for esophageal varices using color Doppler, the indications of EUS-guided vascular interventions have been growing ever since [4,5,6,7,8].

As the world of EUS-guided procedures is developing rapidly, every day, new studies emerge, describing their safety and efficacy in the context of vascular indications. Hence, in this review, we aim to discuss these recently published clinical studies to understand the advancement in the field of EUS-guided vascular interventions (Table 1 and Table 2).

## 2. EUS-Guided Treatment of Gastric Varices

**Figure 1:**
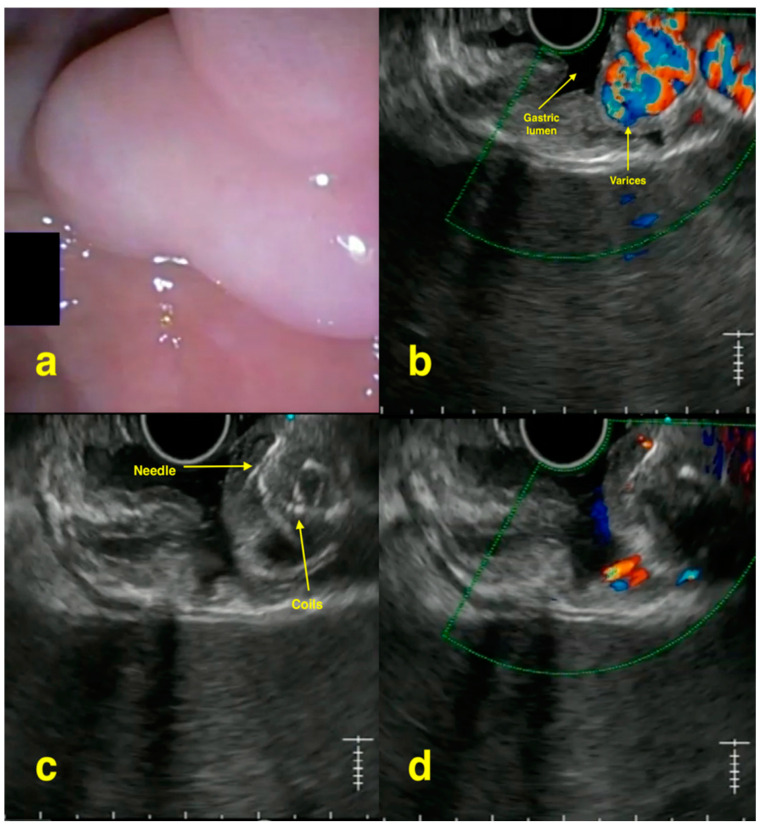
EUS-guided gastric variceal obliteration: (**a**) endoscopic image showing isolated gastric varices; (**b**) EUS image with Doppler showing varices with flow; (**c**) varix punctured with 19 g needle and coils being deployed; (**d**) EUS image with Doppler showing complete obliteration of the varices with coil–glue cast.

Gastric varices, present in around 20% of patients with cirrhosis, are less common than esophageal varices but cause more severe bleeding and worse patient outcomes [8]. The approach to management of gastric varices can be divided into primary prophylaxis (found during surveillance endoscopy), treatment of acute bleeding episodes, and secondary prophylaxis (to prevent rebleeding) [8]. While endoscopic injection of varices with cyanoacrylate (CYA) glue under direct visualization is the most commonly used technique for achieving hemostasis, some gastric varices in the cardia and fundus regions, such as isolated gastric varices type 1 and gastroesophageal varices type 2, are difficult to manage due to their size and location [7]. The advantages of EUS-guided therapy include (a) real-time visualization of the varices including optimum information about their size number as well as the presence of significant perforator/feeder vessels; (b) coil, glue or a combination of the two, which can be used to achieve variceal obliteration. This combination has the advantage of the coil acting as a scaffold for the glue. Figure 1 shows an example of EUS-guided treatment of gastric varices. This prevents glue embolization and lowers the amount of glue required. Finally, (c) assessment of the obliteration is more objective rather than just the feeling of “hardness” upon probing [16].

### 2.1. Primary Prophylaxis

For primary prophylaxis of gastric varices, the American Association for the Study of Liver Diseases (AASLD, 2024) recommends consideration of non-selective beta blockers and endoscopic CYA injection [8]. A recent randomized controlled trial (RCT) by Sabry et al. (2023) reported a higher variceal obliteration rate during the index session (77.2% vs. 38.1%, *p* = 0.014), a smaller amount of CYA needed (1 vs. 2 mL, *p* = 0.027), and a similar rate of overall adverse events (4.5% vs. 14.3%, *p* = 0.345) with EUS-guided CYA injection into the perforating veins vs. direct endoscopic injection [9].

### 2.2. Secondary Prophylaxis

Many newer studies have included patients with both active variceal bleeding and secondary prophylaxis, with enrolment of only a few patients needing primary prophylaxis. In a retrospective study of 80 cirrhotic patients with large gastric varices by Jamwal et al., endoscopic glue injection was compared with EUS-guided therapy (coiling and glue) [17]. The EUS group had lower number of mean sessions needed (1.2 vs. 4), glue volume required (2 ± 0.91 vs. 6 ± 2.31 mL), complications (5 vs. 12), rebleeding events (0 vs. 5), and rescue procedures (0 vs. 3 balloon occlusion with retrograde transvenous obliteration [BRTO]). A similar retrospective study in China showed 100% technical success with both EUS-guided coil plus CYA injection and conventional endoscopic CYA injection of gastric varices with spontaneous portosystemic shunts (SPSSs) [18]. The former treatment modality was superior in terms of lower volume of CYA used (1.64 ± 0.67 vs. 2.38 ± 0.72 mL, *p* < 0.001) and late rebleeding rate (4.8% vs. 27.8%, *p* = 0.041).

Addressing the relatively small sizes of other studies, a recent international multicenter propensity-matched analysis by Samanta et al. compared EUS-guided therapy (coils and CYA glue, n = 58) with 118 cases of endoscopic CYA injection in patients with gastric varices [10]. Replicating previously reported findings, this study also showed better outcomes in the EUS group with respect to lower number of sessions needed (1 vs. 1.5, *p* < 0.0001), re-bleeding episodes (13.8% vs. 39.1%, *p* < 0.0001), and re-intervention rates (12.1% vs. 50.4%, *p* < 0.001). Furthermore, the varix size and use of endoscopic CYA injection were found to be significant predictors of re-bleeding in multivariable regression analysis.

Given that most of the studies have been conducted in the eastern countries, Bazarbashi et al. (2024) reported the first US multicenter retrospective analysis of EUS-guided coil injection of gastric varices [11]. In this cohort of 106 patients, EUS-guided coil injection (with adjunctive glue or absorbable gelatin sponge in 82% patients) achieved a technical success rate of 100% and clinical success rate of 88.7%. Adverse event rates were low (intraprocedural 1.8% and postprocedural 4.7%). Around 14.1% of patients experienced recurrent bleeding, of which 80% were successfully treated with repeat EUS procedure.

Strengthening this evidence further, RCTs have also shown favorable outcomes with EUS-guided treatment of gastric varices. In an RCT by Wang et al. (2023), EUS-guided CYA injection of type 1-isolated gastric varices was shown to be better than direct endoscopic injection of CYA in terms of number of sessions needed (*p* = 0.005), late rebleeding rate (6.7% vs. 22.7%, *p* = 0.032), and the rate of postinjection ulcers (8.9% vs. 27.3%, *p* = 0.023) [12].

Several new iterations and modifications of EUS-guided variceal treatments are currently being evaluated. In an observational study of 27 patients with gastric variceal hemorrhage due to left-sided portal hypertension, the EUS-guided selective N-butyl-2-cyanoacrylate (NBC) injection group was associated with reduced NBC doses (2 vs. 3.1 mL, *p* = 0.004) without any difference in technical/clinical success rates, complications, and rebleeding rates when compared with conventional endoscopic NBC injection group [19]. In another multicenter study, Huang et al. compared EUS-guided coiling/CYA injection and BRTO for gastric varices with high risk of ectopic embolism (SPSS) [20]. Both the groups were comparable in terms of technical success (96.6% vs. 95.6%, *p* = 1.000), 1 year rebleeding rate (20% vs. 18.9%, *p* = 0.900), 1 year mortality rate (2% vs. 0%, *p* = 1.000), and duration of hospitalization (16 vs. 16.5 days, *p* = 0.165). Similar outcomes were reported in the case series by Tang et al. [21]. Going a step further, Xiao et al. explored the utility of combined EUS-guided coiling/CYA injection and BRTO in a patient with gastric varices and huge gastrorenal shunt, achieving complete obliteration without major complications [22].

As thrombin, a liquid embolization agent, is commonly used in IR procedures, O’Rourke et al. applied it in conjunction with EUS-guided coil placement for managing gastric varices [23]. The thrombin/coil combination resulted in a 95% technical success rate, with 85% of patients achieving obliteration of flow within the varices and only 2/20 patients having rebleeding, which was successfully treated endoscopically. In another study of three patients with gastric varices undergoing EUS-guided therapy, Nagashima et al. substituted commonly used wool coils with larger 0.035 inch hydrocoils, which allowed for pull back, smooth deployment, and a stronger blockade of blood flow due to their larger size and hydrogel swelling inside [24]. All three cases had technical success with full obliteration and no adverse events during follow-up.

### 2.3. Primary vs. Secondary Prophylaxis

Most of the data on the EUS-guided approach concern the treatment or secondary prophylaxis of gastric varices. Sarkis et al. compared clinical outcomes in EUS-guided primary (n = 24) and secondary prophylaxis (n = 95) of gastric varices [25]. With the expected lesser stigmata of variceal bleeding (0 vs. 49.5%, *p* < 0.001) and amount of CYA injected (1.4 vs. 1.8 mL, *p* = 0.02) in the primary prophylaxis group, the two groups were comparable in terms of adverse events (25% vs. 23.2%, *p* = 0.8) and post-treatment bleeding rate (0 vs. 7.8%, *p* = 0.33).

### 2.4. Cumulative Evidence

Chandan et al. conducted a meta-analysis of 18 studies (604 patients) to explore the pooled outcomes of EUS-guided management for primary and secondary prophylaxis of gastric varices; the observed obliteration rates were 90.2% and 83.6%, and post-therapy bleeding rates were 4.9% and 18.1%, respectively [13]. In another meta-analysis of 17 studies evaluating the therapeutic role of EUS in liver diseases, Gadour et al. reported EUS-guided treatment of gastric varices to have high technical success and variceal obliteration rates (98% and 84%), with low rates of complications (15%) and rebleeding events (17%) [26]. Using the surface under the cumulative ranking curve (SUCRA), a network meta-analysis of 34 studies (2783 patients) recently showed highest variceal obliteration and lowest rebleeding rates with BRTO (SUCRA 95.1 and 85.1), followed by EUS-guided coiling with CYA injection (SUCRA 80.9 and 78.8) [27]. Interestingly, the endoscopic thrombin injection group had the lowest rate of moderate-to-severe adverse events (SUCRA 92.5), with the mortality being lowest in the EUS-guided coiling with CYA injection group (73.5).

### 2.5. Guidelines

With the overwhelming newer evidence in support of interventional EUS in patients with gastric varices, upgrading its position in the treatment algorithm may be warranted across all national societies [28,29]. The 2021 Baveno VII consensus mentioned EUS-guided therapy with tissue adhesive ± coils in its research agenda for patients with acute variceal bleeding [30]. Currently, the AASLD 2024 guidelines have placed endoscopic coiling as an option in addition to endoscopic CYA injection for bleeding gastroesophageal varices type 2/isolated gastric varices or ectopic varices in centers with local expertise [8].

### 2.6. Other EUS Uses

Adding to the role of EUS in this subset of patients, Wang et al. studied the EUS-guided CYA injection combined with application of Indian ink for locating target vessels of gastric varices and compared it with conventional endoscopy [31]. The study found that EUS was able to perform real-time detection of the perforating vessels, and the marker distribution via EUS was significantly different as compared with conventional endoscopy (*p* < 0.001).

## 3. EUS-Guided Measurement of Portosystemic Pressure Gradient (PPG)

The most common pressure indicator for portal hypertension is the indirect measurement of the gradient by IR, known as hepatic venous pressure gradient (HVPG = free-wedged hepatic vein pressure) [7]. Although HVPG values of >10 mm Hg are linked with formation of esophageal varices, with bleeding risk at >12 mm Hg, they bring increased chances of noncirrhotic/presinusoidal portal hypertension misdiagnosis and radiation exposure. EUS-guided PPG measurement is increasingly being adopted by interventional gastroenterologists as it allows direct estimation of hepatic vein portal pressure gradient, obtained using a 25-gauge needle under real time Doppler (EUS-PPG = mean portal pressure–mean hepatic pressure) [32]. While this EUS modality allows for a one-stop endoscopic procedure in cirrhotic patients for pressure calculation, liver biopsy, and variceal evaluation, the only caveat to using EUS in this setting is the need for general anesthesia to increase stability during the procedure.

Among the newly published literature, Romero-Castro et al. conducted a case study of 21 patients, in which EUS-guided PPG was successful in 90% with the average procedure time being 24 ± 12 min [33]. No major adverse events occurred, except for one patient who had transient epigastric pain. Dhindsa et al. performed the first meta-analysis on EUS-PPG recently, including eight cohort studies (178 patients) [14]. The pooled outcomes reported were technical success in 94.6%, clinical success in 85.4%, total adverse events in 10.9% (93.7% mild, 11% abdominal pain, 3.6% bleeding), with no cases of perforation or death. Even though the standard HVPG measurement technique also has a high technical success rate and a low risk of complications, the above-mentioned limitations make EUS-PPG a potentially superior modality, necessitating a direct comparison study to evaluate the differences between the two techniques [34].

Several factors do need to be kept in mind while assessing the role of EUS-guided PPG measurement in portal hypertension patients. First, simultaneous deep variceal evaluation, liver biopsy, and variceal treatment guided by EUS have not become mainstream yet [35]. Second, contraindications to the EUS approach exist in comparison to conventional HVPG, such as ascites, anatomic anomalies, platelets < 50,000 per microliter or prothrombin time < 50% (<20,000 or <30% for HVPG), and conditions in which upper endoscopy cannot be performed [36,37,38]. Third, given the relatively recent advent of EUS-PPG, measurements need to be validated in the context of clinical outcomes and already established HVPG [7,37]. Fourth, use of anesthetic agents like propofol can lead to alterations of readings while performing EUS-PPG due to their hemodynamic effects.

## 4. EUS-Guided Treatment of Pseudoaneurysms

The clinical presentation of pseudoaneurysms (usually due to inflammation and other etiologies) ranges from being asymptomatic (incidental diagnosis) to major bleeding and are often managed via IR therapy [7]. Jhajharia et al. recently evaluated EUS-guided thrombin injection for pseudoaneurysm management in 20 patients, related to chronic pancreatitis (75%), blunt trauma (15%), recurrent pancreatitis (5%), and idiopathic causes (5%) [15]. Four-to-six weeks post injection, significant improvement was noticed in hemoglobin values (*p* < 0.05), signifying the safety and efficacy of the EUS-guided therapy, especially for areas difficult to reach by IR.

**Figure 2:**
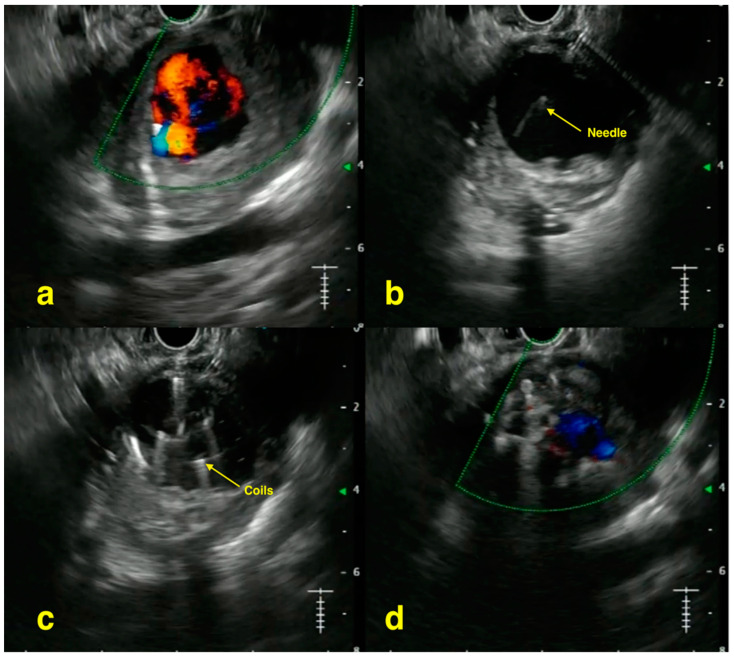
EUS-guided embolization of splenic artery pseudoaneurysm: (**a**) EUS image with Doppler showing splenic artery pseudoaneurysm; (**b**) pseudoaneurysm punctured with a 19 g needle (yellow arrow); (**c**) coils being deployed (yellow arrow); (**d**) EUS image with Doppler showing complete obliteration of the pseudoaneurysm after injecting glue.

Similarly, multiple case reports have also been published. Bouvette et al. showed a case of gastroduodenal artery pseudoaneurysm in a patient with recurrent alcohol-related pancreatitis, which was diagnosed using real-time Doppler with EUS and managed later with vascular coils [39]. In a patient with intermittent bleeding (hemosuccus pancreaticus) due to the rupture of a left gastric artery pseudoaneurysm, Rai et al. demonstrated the utility of EUS-guided glue embolization as the initial IR angioembolization had failed [40]. Samanta et al. successfully used EUS coil plus CYA injection for a patient with likely splenic vessel giant pseudoaneurysm which was not a candidate for IR treatment as it was only visualized during the venous phase of computed tomography (CT) angiography [41]. Another case by Samanta et al. showed the benefit of power Doppler in locating a pseudoaneurysm among multiple collateral vessels in the peripancreatic space, which was subsequently treated with CYA injection [42]. Lastly, Ogura et al. reported a patient who was initially thought to have intraductal tubulopapillary neoplasm based on imaging; however, contrast-enhanced EUS later revealed a splenic artery aneurysm which likely had bled transiently with spontaneous hemostasis [43]. The aneurysm was later treated via an endovascular approach. Figure 2 depicts a case of EUS-guided embolization of splenic artery pseudoaneurysm.

## 5. EUS-Guided Embolization of Splenic Artery

IR-guided partial splenic artery embolization (PSE) in patients with cirrhosis and hypersplenism can help in decreasing portal pressure and its associated complications [7]. EUS-guided PSE via a transgastric puncture has been shown to be a viable alternative to the IR route, which typically involves femoral artery puncture and carries the risk of radiation and post-embolization syndrome [44]. Case reports have shown good success with EUS-PSE, which would need larger studies to compare with IR therapy and to evaluate the effect on overall patient outcomes [44,45,46].

## 6. EUS-Guided Treatment of Other Varices

Varices in other locations such as rectal, ectopic, and parastomal may occur in portal hypertension patients, with the AASLD recommending management similar to that for gastric varices [8]. In a patient with refractory hepatic encephalopathy who was found to have rectal varices, EUS-guided sclerotherapy led to a decrease in ammonia levels and improvement in symptoms [47]. Another patient with pancreaticojejunal (ectopic) variceal bleeding underwent successful obliteration through EUS-guided injection of histoacryl and lipiodol, with no recurrent bleeding over a 1.5 year follow-up [48]. Parastomal varices arising from conjunction of splanchnic veins with the abdominal wall systemic veins can cause repeated bleeding episodes [49]. In a retrospective study of 24 patients with parastomal varices, Todd et al. reported technical success of 100% with EUS-guided thrombin injection ± coil placement [50]. Over a 26.2-month follow-up period, after one procedure, 70.8% patients did not have any additional episodes of significant bleeding, and no major procedure-related adverse events were observed.

## 7. EUS-Guided Management of Non-Variceal Bleeding

Non-variceal upper GI bleeding due to ulcers, tumors, Dieulafoy’s lesions, hemosuccus pancreaticus, and pseudoaneurysms can be challenging to treat due to their variable severity and location [3,51,52]. Despite the standard treatments such as endoscopic drug injections (CYA, epinephrine), topical treatment and thermal coagulation, rebleeding and refractory cases of non-variceal etiologies are still common [51]. In a case series of 17 patients with non-variceal bleeding who were poor candidates for or failed other treatments, EUS-guided hemostasis was performed, which led to 88% patients having no rebleeding over a 12-month follow-up [53]. Similarly, Uribarri-González et al. conducted an observational study of 14 patients with bleeding etiologies identified as Dieulafoy’s lesions/submucosal tumors [54]. A 78% success rate (no rebleed) was observed with EUS-guided injection of polidocanol or 99% ethanol in this patient cohort, who were refractory to previously tried standard endoscopic treatments.

## 8. Miscellaneous Applications

SPSSs are formed in patients with cirrhosis and are a risk factor for developing hepatic encephalopathy. In a case series by Rathi et al., EUS-guided transgastric shunt obliteration (ETSO) was performed via injection of glue/coils into the SPSS [55]. Resolution of encephalopathy without recurrent events over 1 month and complete obliteration was achieved in 6 out of 7 patients. In another recent case of cirrhosis by Rathi et al., EUS-guided porto-splenic split (EPSS) was performed, where the splenic vein was occluded near the portal vein, thereby preventing the mesenteric blood shunting into the systemic circulation [56]. This led to improvement in the patient’s hepatic encephalopathy, with no further symptoms even at 3-month follow-up.

Kotinda et al. showed a case of successful identification of portal cavernoma cholangiopathy (PCC) via EUS in a patient with chronic portal vein thrombosis due to human immunodeficiency virus infection [57]. Feng et al. published a case of a giant esophageal hemangioma, where EUS was used for evaluation, diagnosis (fine needle aspiration), and management (sclerotherapy with lauromacrogol) [58]. EUS-guided portal vein sampling of circulating tumor cells in patients with suspected pancreatic cancer can provide a greater detection rate compared to peripheral blood and has lesser complication risk compared to surgical sampling [59]. Finally, EUS-guided intrahepatic portosystemic shunt placement has been tested in preclinical studies to decompress the portal system along with direct measurement of portal pressure [7]. Other interventions such as EUS-guided thrombolysis of portal vein thrombosis have shown good technical success rates in case series [1].

## 9. Status as per Guidelines

Guidelines from various national societies have differed regarding the role of interventional EUS, largely due to the conflicting data in clinical studies. The European Society of Gastrointestinal Endoscopy (ESGE, 2022) guidelines mention that EUS-guided prophylaxis or management of esophagogastric variceal hemorrhage using coil plus CYA injection may be considered on an individual basis and limited to centers with expertise in this technique (weak recommendation, low-quality evidence) [6]. The 2023 commentary by the American Gastroenterological Association (AGA) on interventional EUS for vascular investigation and therapy suggested that (1) EUS-guided gastric variceal therapy is safe and effective but needs larger comparative studies to define its place in the treatment algorithm; (2) EUS-guided PPG measurement is best considered if endoscopy is being performed for another indication, such as liver biopsy or variceal screening; and (3) due to the limited data available, further evidence is needed in support of other EUS-guided interventions such as splenic artery embolization, portal sampling, and treatment of rectal/ectopic varices and arterial bleeding [7]. The latest practice guidance from the AASLD mentioned that even though EUS can be used for PPG measurement, it is usually estimated indirectly via a transjugular catheter placed in the hepatic vein [8].

## 10. Conclusions

Among the recent advances in EUS [60,61,62], EUS-guided vascular interventions represent a very promising field. Various substances with different properties are available for these interventions, such as glues, fibrin, thrombin, coils and Gelfoam [63]. Perhaps the most commonly studied scenario for EUS-guided vascular interventions is gastric variceal treatment, with other applications also gaining momentum on a daily basis. A major observed theme in clinical studies is discussion amongst various specialties in a multidisciplinary team, especially for individual case reports [64,65,66,67,68,69]. While some cases, such as patients with gastric varices, need prompt intervention to control or prevent bleeding, usage of EUS therapies for other vascular indications requires clinical validation first. The availability of interventionalists trained in EUS-guided procedures is another factor, which is likely limited to tertiary/academic centers, as opposed to IR, which is relatively easily accessible [70,71,72,73,74,75,76]. Nevertheless, the substantial benefits associated with EUS indicate a promising future for this field.

## Figures and Tables

**Table 1:** Development of EUS-guided vascular interventions.

Year	EUS-Guided Intervention
2000	EUS-guided sclerotherapy of esophageal varices
2000	EUS-guided treatment of bleeding gastric varices via injection of cyanoacrylate
2008	EUS-guided treatment of non-variceal bleeding (hemosuccus pancreaticus, dieulafoy lesion, duodenal ulcer, gastrointestinal stromal tumor)
2010	EUS-guided treatment of ectopic (rectal) varices
2015	EUS-guided portal vein sampling for tumor cells
2017	EUS-guided measurement of portal pressure gradient

**Table 2:** Relevant recent studies on EUS-guided vascular interventions.

Study	Type	Outcome
Sabry 2023 [9]	Randomized trial	EUS-guided cyanoacrylate (CYA) injection into the perforating veins vs. direct endoscopic injection of gastric varices: higher variceal obliteration rate during the index session (77.2% vs. 38.1%, *p* = 0.014) lesser amount of CYA needed (1 vs. 2 mL, *p* = 0.027) similar overall adverse events rate (4.5% vs. 14.3%, *p* = 0.345)
Samanta 2023 [10]	Propensity-matched analysis	EUS-guided therapy (coils and CYA glue, n = 58) vs. 118 cases of endoscopic CYA injection in patients with gastric varices: lower number of sessions needed (1 vs. 1.5, *p* < 0.0001), re-bleeding episodes (13.8% vs. 39.1%, *p* < 0.0001) and re-intervention rates (12.1% vs. 50.4%, *p* < 0.001)
Bazarbashi 2024 [11]	Retrospective analysis	EUS-guided coil injection of gastric varices (with adjunctive glue or absorbable gelatin sponge in 82% patients): technical success rate of 100% clinical success rate of 88.7% adverse event rates were low (intraprocedural 1.8% and postprocedural 4.7%) around 14.1% of patients experienced recurrent bleeding, of which 80% were successfully treated with repeat EUS procedure
Wang 2023 [12]	Randomized trial	EUS-guided CYA injection of type 1-isolated gastric varices vs. direct endoscopic injection of CYA: lower number of sessions needed (p = 0.005), late rebleeding rate (6.7% vs. 22.7%, p = 0.032), and postinjection ulcers (8.9% vs. 27.3%, *p* = 0.023)
Chandan 2023 [13]	Meta-analysis	EUS-guided management for primary and secondary prophylaxis of gastric varices (18 studies, 604 patients): obliteration rate was 90.2% and 83.6% post therapy bleeding rate was 4.9% and 18.1%
Dhindsa 2024 [14]	Meta-analysis	EUS-guided measurement of portosystemic pressure gradient (8 cohort studies, 178 patients): technical success 94.6%, clinical success 85.4%, total adverse events 10.9% (93.7% mild, abdominal pain 11%, bleeding 3.6%), and no cases of perforation or death
Jhajharia 2024 [15]	Prospective study	EUS-guided thrombin injection for pseudoaneurysm management (20 patients): significant improvement in hemoglobin values (*p* < 0.05)

## Data Availability

The data used in this review paper are available from online databases.

## Abbreviations

EUS	endoscopic ultrasound
GI	gastrointestinal
CYA	cyanoacrylate
AASLD	American Association for the Study of Liver Diseases
RCT	randomized controlled trial
BRTO	balloon occlusion with retrograde transvenous obliteration
SPSS	spontaneous portosystemic shunts
NBC	N-butyl-2-cyanoacrylate
IR	interventional radiology
SUCRA	surface under the cumulative ranking curve
PPG	portosystemic pressure gradient
HVPG	hepatic venous pressure gradient
CT	computed tomography
PSE	partial splenic artery embolization
ETSO	EUS-guided transgastric shunt obliteration
EPSS	EUS-guided porto-splenic split
PCC	portal cavernoma cholangiopathy
ESGE	European Society of Gastrointestinal Endoscopy
AGA	American Gastroenterological Association
